# An anti-amyloid therapy works for Alzheimer’s disease: why has it taken so long and what is next?

**DOI:** 10.1093/brain/awad049

**Published:** 2023-02-17

**Authors:** John Hardy, Catherine Mummery

**Affiliations:** Reta Lilla Weston Research Laboratories, UCL Institute of Neurology, London, WC1N 3BG, UK; Department of Neurodegenerative Disease, UCL Institute of Neurology, London, WC1N 3BG, UK; Department of Neurodegenerative Disease, UCL Institute of Neurology, London, WC1N 3BG, UK

## Abstract

Hardy and Mummery discuss the recent positive findings in the clinical trial of lecanemab in early Alzheimer’s disease, and the implications for the amyloid hypothesis. They argue that the results mark a turning point for the Alzheimer’s field, but that taking anti-amyloid therapies into clinical practice will be challenging.

The recent announcement of the successful clinical trial of lecanemab (Leqembi) in early Alzheimer’s disease, announced at the Clinical Trials in Alzheimer’s Disease (CTAD) meeting at the end of November 2022, and its recent and rapid FDA approval (https://www.fda.gov/news-events/press-announcements/fda-grants-accelerated-approval-alzheimers-disease-treatment) is a great step forward in the battle against this prevalent disease.^1^ Among those who have seen the data, there has been near unanimous acceptance that the trial, which achieved its primary and secondary end points, is proof that reducing brain amyloid has a clear clinical benefit, consistent with the amyloid hypothesis.^2^ These data stand in contrast to the controversy surrounding the FDA approval of aducanumab (Aduhelm), the previous amyloid removing drug reported, a year earlier.^3^ The reported failure of gantenerumab, a third amyloid removing drug also reported at CTAD was a disappointment. Why has lecanemab worked, why was the aducanumab outcome controversial at best, and why did gantenerumab fail? In fact, the answer to these important questions is largely clear and predictable based on the disease and treatment modelling proposed by Karran and De Strooper.^4^ They suggested (Fig. 1) that the rate and degree of amyloid removal matters, and that clinical benefit accrues once enough amyloid has been removed for a sufficiently long period. Lecanemab effectively removes amyloid and clearly succeeded; of the two aducanumab trials, the one showing greater amyloid removal produced some clinical benefit, whereas the one that showed less removal did not^3^; finally, gantenerumab had disappointing amyloid removal despite its long treatment time and consistent with its non-significant clinical benefits. Clearly, it will be of interest to compare in detail the epitope binding properties of these antibodies to understand both their efficacy profiles and their side effect profiles.^5^

**Figure 1:**
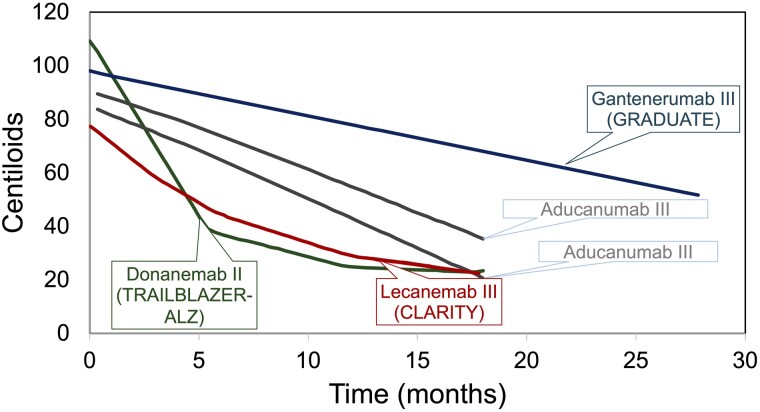
**Amyloid removal by time.** Graphs showing amyloid removal by time in clinical trial updated and redrawn from Karren and De Strooper^4^ and including data from van Dyck *et al*.^1^ and data presented on gantenerumab presented at CTAD. The donenemab data are from phase 2 trials. The other data are from phase 3 trials. The aducanemab 2 trial is the one in which clinical benefit was reported: the aducaneman 1 trial is the one in which clinical benefit was not reported. Clinical benefit seems to become apparent at about the 40 centiloid level.

These are important results because they show what anti-amyloid drugs need to do to be efficacious. They need to reduce brain amyloid significantly and then their trials need to continue long enough after that removal for a clinical benefit to be detectable. It is worth noting that most earlier amyloid antibody trials slowed or prevented amyloid build-up but did not cause amyloid removal^4^ except in the context of the amyloid-related imaging abnormalities (ARIA) treatment complication.

These results mark a turning point for the Alzheimer field. They also pose many challenges ahead.

##  

### The beneficial effects of treatment are real, but modest

The clinical change across primary and secondary end points, relative to placebo, at the end of the 18-month trial was ∼25%,^1^ meaning that those in the treatment arm are at a point equivalent to 15 months in the placebo arm at the end of the trial. At the end of this trial, the treatment and placebo arms appeared to be diverging although both groups were still declining. As part of this debate, it is worth noting that treatment led to significant improvements in caregiver burden. There is already intense discussion about the clinical and economic significance of this benefit. First, is it clinically meaningful? Several months at a higher quality of life is likely to be meaningful to patients and their families; the functional and carer burden trial results would support this. This would lead, for example, to longer independent living and to later admission to nursing home care but would also lead to increased prescription and medical costs. This will, no doubt, be an ongoing and evolving debate because we do not yet know whether longer treatment would achieve greater benefit or whether amyloid build-up would restart, with corresponding loss of efficacy, if treatment was stopped. Examining those that have been on treatment for a number of years will be critical to determining whether there is a cumulative benefit over time.

A very important question that the data raise is what is the pathogenic underpinning of the remaining clinical decline when amyloid is removed? Is the residual decline completely amyloid independent and, if so, is tau/tangle pathology its substrate? A related question is whether can we expect removal of amyloid to ever lead to improvement, or is there a limit to the reduction in decline seen? If we prevent amyloid from accumulating in the first place, can we prevent the onset of disease? That is being tested directly in the upcoming primary prevention trial DIAN-TU-002 (https://clinicaltrials.gov/ct2/show/NCT05269394). These questions will guide future research efforts to completely dissect the underpinnings of neurodegeneration. These will perhaps be best informed by neuropathological investigations of those who have died after being treated after antibody treatment.^6,7^

### Patient selection for treatment will be organisationally challenging

Diagnosing Alzheimer’s disease early and accurately is not easy without the use of biomarkers: for example, a pathological analysis of brains collected by the NIH Alzheimer Centres estimated a diagnostic accuracy rate for Alzheimer’s disease in dementia series of ∼70% with genetic analyses of clinical cases giving similar accuracy estimates.^8,9^ Providing disease-modifying therapies requires a pathologically specific, biomarker confirmed diagnosis. However, our memory services are highly variable in their ability to access diagnostic tests such as CSF analysis or PET scans. Blood biomarkers show great promise; for example, plasma measurement of phospho-tau, which correlates accurately and specifically with plaque deposition, is possibly close to clinical utility.^10^ These would be far easier to integrate into routine clinical practice, potentially enabling a primary care approach provided the right support and criteria are put in place.

### Treatment pathways require a wholesale change in our current services

Memory services are fragmented and under-resourced in the NHS and in most other healthcare systems. They operate primarily on a community-based, palliative level with very few centres equipped to care for individuals having intravenous administration of a drug every 2 weeks, or for the monitoring of potential side effects. Subcutaneous formulations of this drug are under evaluation, which would ease some of the patient and carer burden; however, the hope is that this trial result will prove to be a catalyst for a dramatic overhaul of services, moving towards management of a chronic disease, rather than support for a terminal one.^10^

### The drug side effects require regular monitoring by MRI scan

In the trial, ARIA was monitored by MRI every 3 months. ARIA (focal vasogenic oedematous or haemorrhagic) seems to be a side effect caused by exposure of the amyloid-laden blood vessels to high concentrations of the anti-amyloid antibody. In most cases, ARIA was asymptomatic, had no clinical consequences and administration of the drug was continued. In the majority, the ARIA occurred early in the trial, before the blood vessel amyloid was removed and the problem remitted without clinical consequence. Subsequent to the clinical trial, in the open label phase, two patients died of cerebrovascular events after lecanemab treatment. One of these was on t-PA treatment for ischaemic stroke^10^ and the other on apixaban for atrial fibrillation^11^: it seems likely that these medications contributed to the outcome. These complications in particular show it will be important to monitor and to understand ARIA in terms of pathophysiology and how to predict its occurrence and outcome. Significant additional resources will be needed to ensure safe performance and interpretation of scans is feasible.

### This trial was conducted in those with early symptoms of disease

This trial population had mild symptoms of dementia; however, the pathology has been progressing for decades by this stage, and if we are to optimize the effects of removing amyloid, focussing on earlier stages of disease in future treatment trials is critical. To do that, it is an imperative to organize health systems to enable early diagnosis and in this regard, plasma biomarkers offer perhaps the most likely area where progress could be made.^12-14^

Effective and appropriate delivery of lecanemab will face difficult obstacles in any health system. Now that we know what an amyloid targeting drug needs to achieve to have clinical benefit, it will be easier to develop other drugs, which will deliver benefit, and in this regard, phase 2 data on dononemab (Fig. 1) also look hopeful. It is likely now, that there will be fairly rapid progress relating to -elated treatments. These will include: developing blood biomarkers to aid with diagnosis; formulating amyloid antibodies, which can be administered intravenously; developing therapies and protocols, which minimize or avoid ARIA complications; and testing what the effects are of stopping treatment.

Finally, it seems more than likely that anti-amyloid therapies will eventually be one component of a poly-pharmaceutical treatment regimen for Alzheimer’s disease. We must learn from disease areas who have trodden this road before, such as multiple sclerosis, HIV and cardiovascular disease. Entering a new phase of combination therapies brings with it complexities and more questions, but having one component of such an approach means that trials of drug combinations now have a firm basis from which to start and the experiences of running successful clinical trials for the disease should make the design and execution of such trials easier for both the triallists and the regulatory authorities. A corner has been turned in the attempts to treat Alzheimer’s disease, but there is still some way to go.
